# Host Mesh Fitting of a Generic Musculoskeletal Model of the Lower Limbs to Subject-Specific Body Surface Data: A Validation Study

**DOI:** 10.1155/2019/8381351

**Published:** 2019-02-17

**Authors:** Katja Oberhofer, Silvio Lorenzetti, Kumar Mithraratne

**Affiliations:** ^1^Institute for Biomechanics, Department of Health Sciences and Technology, ETH Zurich, Leopold-Ruzicka-Weg 4, 8093 Zürich, Switzerland; ^2^Swiss Federal Institute of Sport, Magglingen, Switzerland; ^3^The Bioengineering Institute, University of Auckland, Auckland, New Zealand

## Abstract

Challenges remain in accurately capturing the musculoskeletal geometry of individual subjects for clinical and biomechanical gait analysis. The aim of this study was to use and validate the Host Mesh Fitting (HMF) technique for fitting a generic anatomically based musculoskeletal model to 3D body surface data of individual subjects. The HMF technique is based on the free-form idea of deforming geometrically complex structures according to the deformation of a surrounding volumetric mesh. Using the HMF technique, an anatomically based model of the lower limbs of an adult female subject (29 years) was customized to subject-specific skin surface data of five typically developing children (mean age 10.2 years) and six children with Cerebral Palsy (CP) (mean age 9.6 years). The fitted lengths and volumes of six muscle-tendon structures were compared against measures from Magnetic Resonance (MR) images for validation purposes. The HMF technique resulted in accurate approximations of the lower limb shapes of all subjects in both study groups. The average error between the MR data and the fitted muscle-tendon lengths from HMF was 4 ± 4% in children without CP and 7 ± 5% in children with CP, respectively. The average error between the MR data and the fitted muscle volumes from HMF was 28 ± 19% in children without CP and 27 ± 28% in children with CP, respectively. This study presents a crucial step towards personalized musculoskeletal modelling for gait analysis by demonstrating the feasibility of fitting a generic anatomically based lower limb model to 3D body surface data of children with and without CP using the HMF technique. Additional improvements in the quality of fit are expected to be gained by developing age-matched generic models for different study groups, accounting for subject-specific variations in subcutaneous body fat, as well as considering supplementary data from ultrasound imaging to better capture physiological muscle tissue properties.

## 1. Introduction

Computer models of the musculoskeletal system have widely been applied to biomechanical and clinical gait analysis. Musculoskeletal modelling has provided means to quantify muscle and joint function during walking that cannot be measured otherwise. In particular, muscular weaknesses or bilateral asymmetries can result in altered and potentially harmful internal tissue loading which cannot be investigated based on external observation alone. By combining data from optical motion capture with computational models of the musculoskeletal system, crucial insights have been gained into, e.g., muscle-tendon length changes during walking in patients with Cerebral Palsy (CP) to help in the targeted treatment intervention [1], as well as served as intermediate step for calculating muscle-tendon forces and joint loading to assist with rehabilitation intervention and monitoring [2].

Generic musculoskeletal models of the lower limbs have traditionally been adopted and crudely scaled to subject-specific dimensions in order to analyze biomechanical parameters such as joint forces, muscle-tendon lengths, or lengthening velocities during gait for individual subjects [1, 3, 4]. Thereby, the term “generic” refers to a reference model or data set, commonly resembling the anatomy of an adult male or female subject without musculoskeletal injury or disease. In recent years, more advanced optimization algorithms have been introduced in an effort to improve the accuracy of musculoskeletal modelling results for personalized gait analysis [5–8]. Yet, the most widely used fitting algorithms remain based on the positions of bony anatomical landmarks, assuming that the skeletal system sufficiently reflects the subject-specific architecture of the entire musculoskeletal system.

There is growing evidence that the fitting of musculoskeletal models based on bony anatomical landmarks may lead to incorrect conclusions, especially for clinical gait analysis in patients with severe musculoskeletal impairments due to conditions such as CP. Muscle architecture has been found to be significantly altered due to CP [9–11], and bone deformities, commonly observed in children with CP, have been shown to significantly affect joint kinematics, muscle-tendon lengths, and muscle moment arms during walking [12, 13]. Furthermore, bone deformities in the distal segments have been related to altered joint kinematics in the proximal joints and vice versa [14], and changes in the path of one muscle-tendon structure may affect the paths of neighboring muscles and hence the dynamics of the entire multibody musculoskeletal system. Such local differences in musculoskeletal architecture cannot be captured using generic musculoskeletal models that are simply scaled based on the positions of bony anatomical landmarks.

Magnetic Resonance (MR) and ultrasound imaging provide additional insights into the musculoskeletal architecture of individual subjects and have been considered for application to clinical gait analysis. Novel algorithms have been developed to automatically segment MR images based on previous knowledge from generic image data sets [15, 16]; and fitting techniques have been introduced to morph generic models of individual organs to a limited number of subject-specific MR images [17–19]. Yet, the implementation of image-based fitting algorithms to widespread clinical practice has often been a challenge due to long acquisition times of MR imaging as well as high imaging and computational costs. The integration of ultrasound imaging to gait analysis is considered more feasible; yet, ultrasound imaging is confined to a small imaging field of view, e.g., calf muscles, and thus requires additional means of fitting the entire multibody musculoskeletal system to individual subjects [20].

The aim of this study was to use a free-form deformation technique known as Host Mesh Fitting (HMF) for fitting generic musculoskeletal models to 3D body surface data of individual subjects and assess its accuracy in an effort to address the persisting limitations in musculoskeletal modelling for personalized gait analysis. The HMF technique was initially introduced to develop subject-specific 3D models of individual organs [18] and was later applied and validated for predicting the deformation of muscle-tendon structures in the lower limbs during walking [21]. The present work extends on these previous efforts by aiming to fit a generic musculoskeletal model of the lower limbs of an adult female subject to 3D body surface data of children with and without CP and compare the fitted lengths and volumes of six muscle-tendon structures with the subject-specific muscle-tendon lengths and volumes derived from MR data. The use of 3D body surface data and HMF for fitting musculoskeletal models to individual subjects is expected to be particularly suited for gait analysis in population groups where bony anatomical landmarks are not sufficiently accurate and MR scanning not applicable due to time, cost, or ethical constraints.

## 2. Materials and Methods

### 2.1. Volumetric Host Mesh Fitting

**Figure 1:**
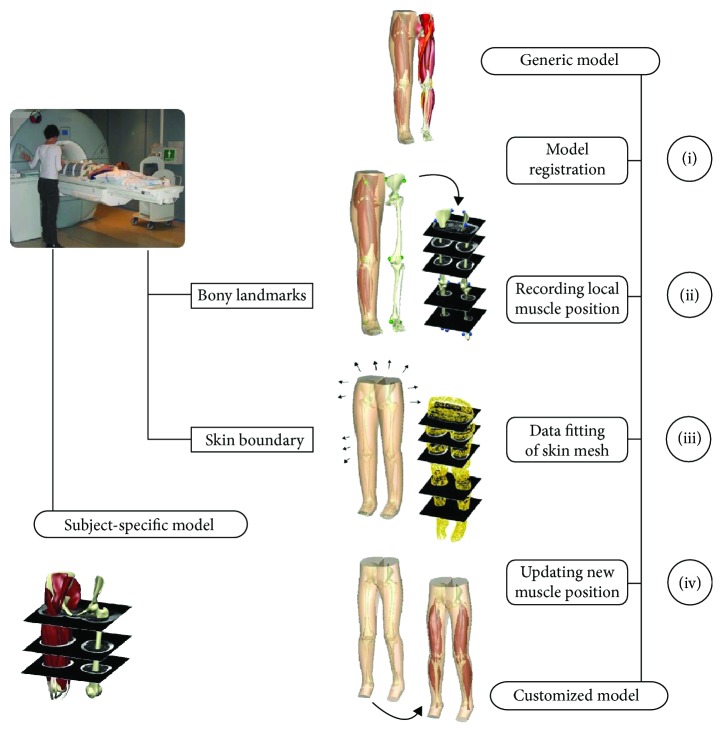
The HMF technique is divided into four steps: (I) model registration based on bony landmarks; (II) recording of muscle-tendon nodal parameters with respect to generic lower body mesh; (III) customization of lower body mesh to subject-specific body shape data; and (IV) updating muscle-tendon nodal parameters according to customized skin mesh. Previously acquired MR images of children with and without CP [11] were used for validation purposes in the present work.

In the following, the theoretical principles of the HMF technique are summarized. Given a generic 3D model of the musculoskeletal system with embedded tissue structures (e.g., muscles-tendon structures and bones of the lower limbs) and subject-specific skin surface data, the HMF process is divided into four steps (Figure 1).

In Step I “Model registration,” the generic lower body mesh is aligned and homogenously scaled to subject-specific dimensions by calculating an overall affine transformation matrix using the conventional positions of bony anatomical landmarks. The affine transformation matrix comprises rotation, scaling, shearing, and translation and is obtained by minimizing the distances between bony anatomical landmarks of the generic model and manually annotated subject-specific bony landmarks. In Step II “Recording local muscle position,” the material positions of the muscle-tendon structures of the generic model are calculated with respect to the surrounding 3D lower body mesh in preparation for skin mesh fitting. In Step III “Data fitting of skin mesh,” the registered lower body mesh is customized to subject-specific 3D body surface data to find the optimum mesh nodal degrees of freedom (i.e., mesh nodal parameters, including nodal positions as well as nodal derivatives in the case of bicubic-linear interpolation functions). In brief, the HMF objective function *F*(**u**_*n*_) is set up to find the optimum mesh nodal parameters **u**_*n*_ that minimize the Euclidean distances between the subject-specific data points and their projections onto the lower body mesh in a least-square sense as follows:

(1) $$\begin{array}{c} F\left(u_{n}\right)=\overset{D}{\underset{d=1}{\sum }}{\left(\overset{N}{\underset{n=1}{\sum }}\left[\varphi_{n}\left(p_{d}\right)u_{n}\right]-s_{d}\right)}^{2}+\delta \left(u_{n},\gamma_{i}\right), \end{array}$$

whereby *p*_*d*_ denotes the coordinates of the projection points *d* = 1,…, *D* with respect to the lower body mesh, and *s*_*d*_ is the corresponding global coordinates of the subject-specific target points, and *δ*(**u**_*n*_, *γ*_*i*_) is a 3D smoothing constraint called Sobolev function with user-defined penalty parameters *γ*_*i*_ ∈ [0, 1] for controlling arc lengths, curvatures in element coordinate directions, surfaces area terms, and volume of the lower body mesh. Further details to the HMF objective function and 3D smoothing constraints can be found in [18, 21]. Finally, in Step IV “Updating new muscle position,” the spatial positions of the muscle-tendon structures are calculated according to the customized position of the lower body mesh. This is carried out under the assumption that the material positions of the muscle-tendon structures with respect to the surrounding 3D lower body mesh do not change during customization.

The HMF algorithm is implemented in the modelling environment CMISS (http://www.cmiss.org). CMISS is an interactive computational modelling environment for Continuum Mechanics, Image analysis, Signal processing, and System identification, which has extensively been used for high-order subject-specific modelling of the musculoskeletal system [11, 18, 21, 22]. CMISS has been developed as part of the International Union of Physiological Sciences (IUPS) Physiome Project [17, 19] and is currently being redeveloped into the open source package Open-CMISS to make it more modular, extendable, easier to understand, and able to run on modern distributed-memory high-performance computers (http://www.opencmiss.org).

### 2.2. Validation

The accuracy of the HMF technique was assessed by fitting a generic lower limb model to subject-specific data of children with and without CP and comparing the predicted muscle-tendon lengths and volumes of the fitted model with subject-specific MR data. A generic lower limb model of an adult female subject, which was previously manually developed based on subject-specific MR data [21], was used (age 29 y, height 165 cm, and weight 63 kg) for this purpose. The lower limb model comprised all lower limb bones, 20 muscles-tendon structures, and a volumetric representation of the skin boundary surface of each leg. All geometries of the musculoskeletal lower limb model were represented using high-order finite element meshes with bicubic-linear interpolation functions. Cubic interpolation functions preserve the continuity of the first derivatives of the geometric coordinates with respect to the element coordinates, which makes them ideal for smoothly approximating the curved surfaces of biological tissue with a minimum number of elements [21].

MR images of the lower limbs of six children with CP (mean age 9.6 years) and five typically developing children (mean age 10.2 years) were acquired on a Siemens 1.5T MAGENTOM Avanto System. Ethical approval was given by the NZ Northern Y Regional Ethics Committee, reference number NTY/06/07/064. Written consent was obtained from all children and their parents or guardians. Subject characteristics and scan protocol have previously been outlined in detail [11]. The image processing tools within CMISS were employed to automatically segment the skin boundary surfaces of the lower limbs. The positions of the following bony landmarks on the skin surface were manually identified according to standard protocols [23]: right/left asis, sacrum, medial/lateral epicondyles, and medial/lateral malleoli. The bony landmarks were used to register the generic model to the subject-specific surface data (Step I, Figure 1). The lower body mesh was then customized to subject-specific skin surface data and the new configuration of each muscle-tendon structure was calculated according to the customized lower body mesh (Steps II-IV, Figure 1).

Muscle-tendon lengths and volumes of the fitted models were numerically derived and compared with subject-specific measures from MR images for validation purposes. The following six muscles were included in the analysis: soleus, gastrocnemius, semimembranosus and semitendinosus (represented as one muscle), biceps femoris, and the vasti group. Muscle-tendon lengths were defined as the average arc lengths between the most distal and most proximal ends of the muscle-tendon meshes, normalized with respect to segmental lengths. Muscle volumes were derived by performing numerical quadrature over the parameterized meshes (Fernandez et al., 2005), divided by body mass. The fitting error *E*_HMF_ was defined as the relative difference in muscle-tendon length *l*, i.e., muscle volume *V*, between the fitted values from HMF and the subject-specific values derived from the MR images:

(2) $$\begin{array}{c} E_{\text{HMF}}^{l}=\frac{\left|l_{C}-l_{\text{MRI}}\right|}{l_{\text{MRI}}},\\ E_{\text{HMF}}^{V}=\frac{\left|V_{C}-V_{\text{MRI}}\right|}{V_{\text{MRI}}}. \end{array}$$

### 2.3. Statistical Analysis

Statistical analysis was performed to assess the significance of the differences in muscle-tendon lengths and volumes between the fitted and the subject-specific values from MR imaging. All parameters were tested for a normal distribution prior to data comparison using the Kolmogorov and Smirnov method [24]. A repeated measure analysis of variance (ANOVA) with Tukey-Kramer multiple post hoc test [25] was performed to analyze the pairwise differences in muscle-tendon lengths and volumes between the fitted and the subject-specific measures from MR imaging. The data of the children with CP and without CP were analyzed independently as two different groups. Statistical analysis was performed using the statistical software GraphPad IntStat. The level of significance was set at *p* < 0.05 for all statistical test.

## 3. Results

**Figure 2:**
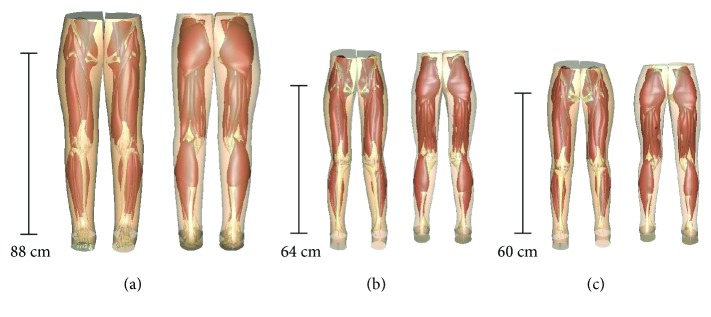
(a) The generic lower limb model based on the anatomy of an adult female (Oberhofer et al. 2009). (b) A fitted lower limb model of a child without CP. (c) A fitted lower limb model of a child with CP. Leg lengths of each model, ranging from the hip joint center to the ankle joint center, are given as reference.

A generic lower limb model of an adult female subject was fitted to skin surface data of children with and without CP using the HMF technique. Eleven bony landmarks and an average number of 1,858,218 (±845) data points on the skin boundary of each subject were used for the fitting process. The HMF technique resulted in smooth approximations of the lower body shapes of all subjects in both study groups (Figure 2). The average Root Mean Square (RMS) error between the fitted lower body mesh and the subject-specific surface data from MR imaging was 3.7 ± 1.08 mm.

The average normalized muscle-tendon lengths derived from HMF compared to the subject-specific values from MR images are given in Table 1. Statistical analysis revealed that HMF led to accurate predictions of muscle-tendon lengths in the children without CP for all muscles except rectus femoris. In the children with CP, HMF led to accurate predictions of muscle-tendon lengths for soleus, biceps femoris, and the vasti group, while significant differences were obtained between the fitted and the MR-based values for gastrocnemius, semimembranosus-semitendinosus, and rectus femoris. The average fitting error (equation (2)) in muscle-tendon lengths from HMF was 4 ± 4% in the group of children without CP and 7 ± 5% in the children with CP, respectively.

The average normalized muscle volumes derived from HMF compared to the subject-specific values from MR images are given in Table 2. Overall, the prediction of muscle volumes was poor, with an average fitting error (equation (2)) of 28 ± 19% in children without CP and 27 ± 28% in children with CP, respectively. Statistical analysis revealed significant differences in the predicted muscle volumes from HMF compared to MR imaging for four muscles in the children without CP (soleus, biceps femoris, rectus femoris, and vasti group) and for two muscles in the children with CP (biceps femoris, semimembranosus-semitendinosus).

## 4. Discussion

The aim of this study was to address current limitations in subject-specific musculoskeletal modelling for personalized gait analysis by applying and validating the HMF technique to fit a generic model to subject-specific 3D body surface data. The HMF technique extends scaling of generic musculoskeletal models based on bony anatomical landmarks in that it comprises an affine transformation (rotation, translation, and scaling) followed by model customization to account for subject-specific variations in lower limb shape. High accuracies were obtained in the fitted lower limb shapes in both study groups with the RMS error between the subject-specific 3D body surface data and the fitted lower limb mesh being less than 5 mm for all data points. The accuracies in muscle-tendon lengths are also considered promising for having the potential to improve gait analysis results, with an average RMS error of 4 ± 4% in the children without CP and 7 ± 5% in the children with CP, respectively (Table 1). The average RMS errors in muscle-tendon lengths in both study groups are below, or around the lower range, of previously reported errors in muscle-tendon length predictions using generic musculoskeletal models for clinical gait analysis, e.g., 6% to 50% [26]. However, the accuracies in muscle volumes were limited with large variations in both study groups compared to the subject-specific MR data (Table 2).

**Figure 3:**
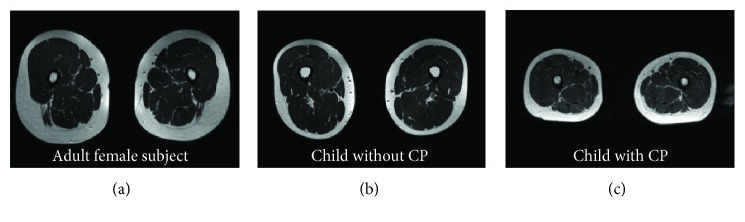
Representative MR image of the shank of (a) adult female subject, (b) child without CP, and (c) child with CP.

The HMF technique is established under the assumption that the lower limb shape reflects the internal musculoskeletal architecture, which is a limitation of the proposed technique. It means that the relative positions of muscle-tendon structures with respect to the skin mesh remain constant during model fitting. If, for example, a thick subcutaneous fat layer between muscles and skin is present in the generic model, the relative thickness of the fat layer remains the same throughout HMF. Looking more closely at the MR images (Figure 3), it becomes apparent that significant differences existed in muscle volumes between individual subjects. In particular, children subjects had less subcutaneous fat compared to the adult female subject, which could partly explain the unsatisfying prediction of muscle volumes compared to muscle-tendon lengths. Interestingly, the average RMS error for muscle volumes was slightly lower for the children with CP than the children without CP, which is an unexpected result (Table 2). Based on the MR images (Figure 3), it appears as if the percentage of muscle tissue versus fat tissue in children with CP more closely resembled the adult female anatomy, e.g., thicker fat layer with less muscle tissue, which may explain the unexpected outcome. Thereby, the volumetric tissue distribution critically affects the inertia properties of the multibody dynamic system and hence gait analysis results. These insights suggest that additional skin fold measurements may help to improve model fit by allowing to adjust the relative thickness of the fat layer, and thus segmental inertia properties, for individual subjects.

The time needed to develop musculoskeletal models by manually segmenting MR images is lengthy and can take several months. Currently, the modelling software CMISS contains a library of MR-based lower limb models of one female subject, six children with CP, and five typically developing children, which were adopted in the present work. The present goal to accurately fit a generic model of an adult female subject to the anatomy of children with severe gait impairments due to CP was ambitious. It is likely that more accurate results can be obtained when fitting the generic model to subjects of similar age and without significant musculoskeletal impairments. Nevertheless, the present results are promising and considered the first step towards an advanced modelling framework for subject-specific simulation and analysis of human movement. In addition to the MR-based lower limb models within CMISS, data from gait analysis was acquired in the same subjects. This unique dataset will allow the comparison of muscle-tendon length calculation during walking between generic and HMF-fitted musculoskeletal models as a next step. Furthermore, an extension of the model library based on the Visible Human Dataset from the U.S. National Library of Medicine, which includes Computed Tomography and MR images of one male and one female cadaver, is planned. The Visible Human Dataset has been applied to musculoskeletal research, educational, virtual reality, industry, and diagnostic purposes and thus will provide widely accepted reference models for future use.

The solution of the HMF objective function (equation (1)) is, in the present form, dependent on the geometry of the lower body mesh (i.e., mesh nodal degrees of freedom) and the magnitudes of the Sobolev smoothing constraints. Both, the geometry of the lower body mesh and the Sobolev smoothing constraints, have not been linked to physiological or anatomically based principles but were defined according to previously established kinematic criteria [18]. Kinematic surface-based deformation methods have extensively been used in computer graphics research [27]. Yet, they are traditionally not considering biological soft tissue as elastic solids subject to Newton's laws of motion. In recent work, Kadleček et al. [28] introduced a physics-based model fitting technique to find the optimum shape of a musculoskeletal model based on several 3D body surface scans that minimizes the deformation energy, corresponding to the elasticity of biological soft tissue. The consideration of a so-called elastic potential to find the optimum fit solution (equation (1)) while complying to Newton's laws of motion for soft tissue is promising and may offer the potential to improve the accuracy of the HMF fit for subjects with various degrees of subcutaneous body fat versus muscle tissue.

Additionally, data from ultrasound imaging may allow further insights into mechanical tissue properties to advance the HMF technique based on anatomically aware principles [20]. Capturing subject-specific mechanical properties of soft tissue is particularly important when aiming to analyze kinetic variables, e.g., muscle forces, in patients with musculoskeletal disorders such as CP. Yet, taking subject-specific tissue samples *in vivo* for refining musculoskeletal models remains highly invasive and very compromised. Ultrasound data would allow to better capture mechanical properties of muscles at the tissue level, e.g., physiological cross-sectional area and fiber pennation angle, which in turn affect muscle mechanics. Ultrasound imaging is relatively inexpensive, does not involve ionizing radiation, and requires much shorter scan times compared with other imaging modalities such as MR imaging. Thereby, an anatomically aware deformation method was recently introduced by Saito et al. [29] to predict the growth and size of muscles by discretizing the anisotropic stretch in the direction of muscle fibers. The integration of muscle fiber structures into the present musculoskeletal modelling approach is highly feasible. In particular, a muscle fascicle description has already successfully been integrated into the muscle organ models in CMISS and fitted to subject-specific ultrasound data with good qualitative agreement to diffusion-weighted MR images [30].

In this study, the skin boundary surfaces of individual subjects were segmented based on MR data, though body surface scanning could be used to capture the outer skin surface of individual subjects in future work. Body surface scanning, frequently used in anthropometric body shape analysis and obesity research, offers inexpensive, rapid, and noninvasive means to characterize the skin boundary *in vivo* [31] and would make the application of the HMF technique feasible in clinical settings. Thereby, the numerical algorithms associated with HMF, as well as the library of MR-based musculoskeletal models, are currently transferred into the open source modelling environment Open-CMISS (http://www.opencmiss.org/) to provide the most advanced and accessible numerical tools for physiologically based modelling of deformable organs, e.g., muscle tissue across multiple scales, including multibody dynamic analysis [19, 21, 22, 30].

## 5. Conclusions

The current study presents a crucial step towards personalized human movement analysis by demonstrating the feasibility of fitting a generic musculoskeletal model of the lower limbs to skin surface data of children with and without CP. The musculoskeletal models of the lower limbs and fitting algorithms are planned to be further developed and shared between research centers through the IUPS Physiome Project [19] and coupled with experimentally measured gait data for dynamic simulations of walking. Additional improvements in the quality of fit are expected to be gained by developing age-matched generic models for different study groups, as well as taking into account subject-specific skin fold measures and mechanical properties of muscle tissue based on ultrasound imaging. It is anticipated that the application of personalized musculoskeletal models to movement analysis will lead to crucial new insights into the complex relationship between musculoskeletal architecture and function during dynamic activities and thus assist in the assessment and management of movement pathologies due to conditions such as CP.

## Figures and Tables

**Table 1:** Average normalized muscle-tendon lengths (%) derived from the subject-specific MR images (MRI) compared to the predicted values from HMF for the children without CP and the children with CP.

	Children without CP			Children with CP		
	MRI	HMF	*p* value	MRI	HMF	*p* value
Soleus	71 (5.1)	71 (2.2)	>0.05	66 (4.7)	69 (2.3)	>0.05
Gastrocnemius	60 (5.8)	56 (3.6)	>0.05	51 (3.7)	55 (1.5)	<0.05^∗^
Biceps femoris	58 (4.6)	60 (1.7)	>0.05	54 (3.7)	56 (4.8)	>0.05
Semi group	82 (3.3)	84 (3.7)	>0.05	76 (7.2)	81 (3.7)	<0.01^∗^
Rectus femoris	76 (1.3)	79 (1.6)	<0.05^∗^	68 (3.1)	75 (2.9)	<0.01^∗^
Vasti group	91 (4.1)	91 (5.0)	>0.05	85 (2.1)	86 (2.7)	>0.05

^∗^Difference between MRI and HMF statistically significant (repeated measures ANOVA with Tukey-Kramer multiple Comparison post hoc test, *p* < 0.05).

**Table 2:** Average muscle volumes (cm^3^/kg) derived from the subject-specific MR images (MRI) compared to the predicted values from HMF for the children without CP and the children with CP.

	Children without CP			Children with CP		
	MRI	HMF	*p* value	MRI	HMF	*p* value
Soleus	5.5 (0.84)	3.9 (0.30)	<0.01^∗^	4.5 (1.56)	3.7 (0.51)	>0.05
Gastrocnemius	4.4 (1.01)	3.6 (0.23)	>0.05	3.1 (1.26)	3.6 (0.56)	>0.05
Biceps femoris	2.3 (0.44)	3.5 (0.32)	<0.001^∗^	1.6 (0.41)	3.5 (0.33)	<0.001^∗^
Semi group	5.1 (0.91)	5.0 (0.30)	>0.05	3.9 (0.88)	5.5 (0.51)	<0.01^∗^
Rectus femoris	3.6 (0.69)	2.2 (0.22)	<0.01^∗^	2.6 (0.63)	2.3 (0.63)	>0.05
Vasti group	20.3 (2.80)	16.7 (1.16)	<0.05^∗^	15.9 (3.20)	16.5 (2.58)	>0.05

^∗^Difference between MRI and HMF statistically significant (repeated measures ANOVA with Tukey-Kramer multiple comparison post hoc test, *p* < 0.05).

## Data Availability

The MR image data used for this study are restricted by the New Zealand Northern Y Regional Ethics Committee in order to protect patient privacy. The data is only available to researchers who meet the criteria for accessing the confidential data. Further information can be obtained from the corresponding author Dr. Katja Oberhofer (katja.oberhofer@hest.ethz.ch).
